# The role of Theory of Mind and Empathy levels in Anxiety Disorders Symptomatology

**DOI:** 10.1192/j.eurpsy.2022.979

**Published:** 2022-09-01

**Authors:** G. Santarelli, M. Innocenti, V. Faggi, V. Miglietta, I. Colpizzi, F. Galassi, G. Castellini, V. Ricca

**Affiliations:** 1 University of Florence, Human Health Sciences, Firenze, Italy; 2 University of Florence, Human Health Sciences, firenze, Italy

**Keywords:** Empathy, Theory of Mind, Anxiety disorders, Anxiety

## Abstract

**Introduction:**

Theory of Mind (ToM) is defined as the ability to understand mental states of other people. Recent studies explored its role in various psychopathological disorders, but evidence lacks on the relationship existing between specific psychopathological domains and ToM.

**Objectives:**

We aimed to investigate the relationship between psychopathology of Anxiety Disorders (AD) and Theory of Mind.

**Methods:**

We enrolled 35 patients admitted to the Psychiatric Unit of Careggi Hospital in Florence with diagnosis of AD. We administered them: Zung Anxiety Scale (ZSAS), Metacognition Questionnaire-30 (MCQ-30), and Reading the Mind in the Eyes (RMET). Pearson’s correlation was used to assess relationships between variables.

**Results:**

A significant positive correlation was detected between RMET scores and ZSAS total scores (r=0.385, p=0.022), MCQ-30 Negative Beliefs about Uncontrollability and Danger subscale (MCQ-30-Neg, r=0.407, p=0.015), and MCQ-30 Cognitive Self-Consciousness subscale (MCQ-30-CSC, r=0.349, p=0.040).

**Table:** 

Correlations between the variables in the study and Reading the mind in the eyes total scores are shown.		
	RMET total score	
	r	p
**MCQ-30-Neg**	0.407	**0.015**
**MCQ-30-CSC**	0.349	**0.040**
**MCQ-30 Positive beliefs about Worry**	0.073	0.667
**MCQ-30 Lack of Cognitive Confidence**	-0.245	0.155
**MCQ-30 Need to Control Thoughts**	0.311	0.069
**ZSAS total scores**	0.385	**0.022**

**Conclusions:**

Such preliminary data suggest a relationship between Theory of Mind and AD psychopathology. In particular, some dimensions of AD psychopathology seem to predict higher Theory of Mind levels.

**Disclosure:**

No significant relationships.

